# Prediction of safe driving Behaviours based on health belief model: the case of taxi drivers in Bandar Abbas, Iran

**DOI:** 10.1186/s12889-018-5300-5

**Published:** 2018-03-20

**Authors:** Asghar Razmara, Teamur Aghamolaei, Abdoulhossain Madani, Zahra Hosseini, Shahram Zare

**Affiliations:** 1 Social Determinants on Health Promotion Research Center, Health Institute, Hormozgan University of Medical Sciences, Bandar Abbas, Iran; 2 Department of Health Education and Health Promotion, Health School, Hormozgan University of Medical Sciences, Bandar Abbas, Iran; 3 Department of Social Medicine, Medical School, Hormozgan University of Medical Sciences, Bandar Abbas, Iran

**Keywords:** Health belief model, Safety, Driving, Taxi driver

## Abstract

**Background:**

Road accidents are among the main causes of mortality. As safe and secure driving is a key strategy to reduce car injuries and offenses, the present research aimed to explore safe driving behaviours among taxi drivers based on the Health Belief Model (HBM).

**Methods:**

This study was conducted on 184 taxi drivers in Bandar Abbas who were selected based on a multiple stratified sampling method. Data were collected by a questionnaire comprised of a demographic information section along with the constructs of the HBM. Data were analysed by SPSS ver19 via a Pearson’s correlation coefficient and multiple regressions.

**Results:**

The mean age of the participants was 45.1 years (SD = 11.1). They all had, on average, 10.3 (SD = 7/5) years of taxi driving experience. Among the HBM components, cues to action and perceived benefits were shown to be positively correlated with safe driving behaviours, while perceived barriers were negatively correlated. Cues to action, perceived barriers and perceived benefits were shown to be the strongest predictors of a safe drivers’ behaviour.

**Conclusions:**

Based on the results of this study in designing health promotion programmes to improve safe driving behaviours among taxi drivers, cues to action, perceived benefits and perceived barriers are important. Therefore, advertising, the design of information campaigns, emphasis on the benefits of safe driving behaviours and modification barriers are recommended.

## Background

Traffic accidents and injuries are among the main causes of mortality worldwide [1]. Approximately 1.25 million people die each year as a result of road traffic crashes. Without sustained action, road traffic crashes are predicted to become the seventh leading cause of death by 2030 [2] . Related literature has attested to the key role of safe driving in reducing the rate of driving-induced injuries, offenses and violations [3]. In the Iranian context, the foremost factor involved in the occurrence of car accidents is human factors [4]. The cause of most unsafe acts was attributed to human factors by Heinrich [5].

Considering the existing deficiencies in the public transport system in Iran, taxis enjoy a significant status. In many small or average-sized towns, taxis help to carry out 80% of the town-level transportation [6]. Though often very professional, taxi drivers depend to a large extent on the number of passengers they carry to determine their income. That is why they are pressed to attract more passengers and drive faster than they should, which can tremendously affect their risky behaviours [7]. A body of related research also reported a high rate of risky behaviours, unauthorized speeding, overtaking, stopping mid-street for passengers, giving a lift to passengers while moving, fast and recurrent changing of lanes and unauthorized distance from the car ahead of them [8, 9]. On the other hand, taxi drivers are faced with a high rate of job mortality (14.9%) compared to other jobs (3.3%). Physical violence was shown to be significantly higher in taxi drivers who fail at a day’s work than in other jobs (3.7 vs. 2.4 per 10,000) [10].

Safe behaviours are of the utmost importance when they not only provide for one’s own security and health but also that of others. The majority of events are induced by the drivers’ unsafe behaviours, which makes the recognition and correction of unsafe behaviours more essential. Since prevention is prioritized over treatment, investigations can significantly help to prevent events. Job-related events and safety concerns have a complicated nature and different roots. A prognostic approach seems to be properly fitted to potential solutions [11].

Sociologists, psychologists and anthropologists suggest a range of theories and models to describe the key factors involved in behaviour. Among them is the HBM (HBM), which has been one of the strongest psycho-sociological models commonly used in evaluating the beliefs, values and attitudes towards a wide range of health-related behaviours. The HBM is a psychological health behavior change model developed to explain and predict health-related behaviours, particularly in regard to the uptake of health services [12]. It was developed in the 1950s by social psychologists at the U.S. Public Health Service [13, 14], and remains one of the best known and most widely used theories in health behavior research [15, 16].

This model is comprised of several components: perceived susceptibility, perceived severity, perceived benefits, perceived barriers, cues to action and self-efficacy.Perceived susceptibility refers to subjective assessment of risk of developing a health problem. The HBM predicts that individuals who perceive that they are susceptible to a particular health problem will engage in behaviours to reduce their risk of developing the health problem. Perceived severity refers to the subjective assessment of the severity of a health problem and its potential consequences. The HBM proposes that individuals who perceive a given health problem as serious are more likely to engage in behaviours to prevent the health problem from occurring or reduce its severity. Perceived benefits refer to an individual’s assessment of the value or efficacy of engaging in a health-promoting behavior to decrease risk of disease. If an individual believes that a particular action will reduce susceptibility to a health problem or decrease its seriousness, then s/he is likely to engage in that behavior. Perceived barriers refer to an individual’s assessment of the obstacles to behavior change. Even if an individual perceives a health condition as threatening and believes that a particular action will effectively reduce the threat, barriers may prevent engagement in the health-promoting behavior. Also, the HBM posits that a cue is necessary for prompting engagement in health-promoting behaviours [13–15, 17]. Self-efficacy refers to an individual’s perception of his or her competence to successfully perform a behavior [17] . Self-efficacy was added to the HBM in an attempt to better explain individual differences in health behaviours [18]. According to this model, one begins to act safely only when s/he is assured there is a possibility of accident, which may cause suffering. Once the driver feels threatened, one begins to act to prevent such undesirable events [19].

It is likely that driving behaviours, in particular unsafe behaviours, are partly related to the constructs of the HBM model because previous studies have shown that risk perception as a potential factor that can affect unsafe driving behaviours. The model suggests that the higher risk a behaviour is, the less likely a person is to behave in that manner in the future [20]. In the HBM, the perceived susceptibility and severity can be defined as the perception of personal risk and its behavioural implications. For example, the results of a study by Şimşekoğlu et al. in Turkey showed that the perception of traffic hazards is related to precautionary behaviours, such as using seat belts and driving the speed limit [21]. Use of HBM can be useful in reducing unsafe driving behaviours, such as driving at high speeds. In other words, if the perceived susceptibility and perceived severity of risky driving behaviours increase, the tendency to these behaviours will decrease [22].

Özbay et al. showed that perceived barriers are associated with unsafe driving behaviours, including high speed and driving violations, and changes in perceived barriers are effective in changing the behaviour and reducing driving violations [22]. Additionally, in the study of Lajunen and Räsänen et al., the use of helmets by cyclists was predicted by perceived barriers and cues to action [23] .In the study of Morowatisharifabad et al., HBM constructs, such as perceived susceptibility, perceived severity, perceived benefits, and perceived barriers were significantly associated with risky driving behaviours [24]. In a study by Lajunen and Özkan, entitled driving behaviours of motorcyclists, and their psycho-social reasons, the results showed that the reduction in the perceived barriers was associated with the increased use of safety equipment. In this study, cues to action had an indirect significant relationship with speed violations, and the reduction in the perceived severity construct was associated with a decrease in safety behaviours [25]. In the study of Hatamzadeh et al., HBM constructs were effective in increasing the use of seat belts [26]. Oruogi et al. in a study showed that the perceived susceptibility and perceived barriers were the most important determinants in helmet use [27]. In their research, Tavafian et al. found that the perceived benefits and barriers are the best predictors of wearing seatbelts [28]. The findings of these studies show that the HBM constructs are consistent with the driving behaviours. Although HBM has been used in many studies on health-related behaviours, there is little information about the use of this model to predict safe driving behaviours in taxi drivers.

The application of the theory of planned behaviour (TPB) to predict safety behaviours has been proven in many studies [29–31] and some studies have concluded that this theory is more robust in predicting safety behaviours than the HBM [28, 32]. However, in the current research, HBM was used for several reasons. Taxi drivers think that they are more skilled and experienced than other car drivers and do not consider themselves to be in danger of an accident. On the other hand, some researchers have shown that risk perception can be effective as a potential factor for unsafe driving behaviors [20, 21]. Perceived susceptibility and perceived severity that are essential to preventing high-risk driving behaviours are two main constructs of HBM, while they are not addressed in the TPB. In this study, we intend to predict safe driving behaviors. In this regard, the HBM is also a predictive model. In a meta-analysis, Sheeran and Abraham showed that HBM constructs significantly predict health-related behaviour [33]. Moreover, driving is a part of cultural behaviour of individuals, because it is influenced by the values and beliefs of them. In this regard, the HBM focuses on attitudes and beliefs of individuals and shows the relationship between beliefs and behaviour [34]. Some researchers have concluded that the cultural environment has a significant impact on high risk driving behaviours [21, 35, 36].

In light of the aforementioned body of literature and the existing gap in investigating the taxi drivers’ behaviours, the present study aimed to predict taxi drivers’ safe driving behaviours according to the HBM in Bandar Abbas, a city in southern Iran.

## Methods

### Study population and sampling

The research population was all the taxi drivers in Bandar Abbas. The sample size was determined to be 184, which corresponded to the previous literature [37]. Those who entered the study had worked for at least a year as a taxi driver. They were capable of reading and writing and resided in Bandar Abbas.

The subjects were selected from different taxi stations in the city. According to Taxi Union of Bandar Abbas, there were twenty-four taxi stations in different parts of the city. From this number, eight stations were randomly selected. The number of the required subjects was set according to the approximate number of taxis present in each station. Visits were paid to the selected stations twice a day (morning and evening work shifts), and the sampling was done. The first driver entering each and every station who met the inclusion criteria and was willing to take part would enter the sample. Next, every 6th driver entering the station would be approached for sampling. This procedure went on until the required sample size was met. A total number of 184 questionnaires were submitted to the subjects, and 180 were returned completed in full (response rate = 97.8%).

### Measures

The data collection instrument was a questionnaire that opened with demographic information (age, education, driving experience in general, experience of taxi driving, previous violations and accidents, etc) followed by components of the HBM and safe driving behaviours. The questionnaire was compiled based on the previous body of similar research as well the results of a pilot study. Twelve taxi drivers were involved in a group discussion as a pilot test to explore their comments on the benefits and barriers of safe driving behaviours. The items that explored the HBM constructs were rated on a 5-level Likert scale. The 5 choices included were totally agree, agree, undecided, disagree and totally disagree. The order of the questions was reversed to avoid the halo effect. The items exploring driving behaviours were rated on a 4-level Likert scale ranging from always to never (a score of 1 to 4).

To confirm the validity of the test, a content validation procedure was followed with the help of a panel of experts. To this aim, the questionnaire was availed to 15 drivers that met the research population characteristics. They were asked to comment on the clarity, legibility and relevance of the item. An item would be omitted (or corrected) if more than half of this panel agreed that the question had an improper format or content. As a result, no item was omitted, but some were corrected. The questionnaire was then submitted to a panel of 8 health education and traffic safety specialists and the required revisions were made later on.

Cronbach’s alpha test was used to establish the reliability of the HBM questionnaire and was comprised of the following components:

#### Perceived susceptibility

This component was represented by 5 items, such as “If I drive over the speed limit, I am more likely to crash”. The items were measured on a Likert scale ranging from completely agree (5) to completely disagree (1). The scores of the items were summed up, and the overall scores ranged from 5 to 25. Cronbach’s alpha test for this component was estimated at 0.83.

#### Perceived severity

This component was comprised of 5 items, such as “If I crash when driving, I might die”. The items were measured on a Likert scale ranging from completely agree (5) to completely disagree (1). The scores of the items were summed up, and the overall scores ranged from 5 and 25. Cronbach’s alpha test for this component was estimated at 0.81.

#### Perceived benefits

There were 6 items included within this component, such as “Driving safety reduces the risk of accident”. The items were measured on a Likert scale ranging from completely agree (5) to completely disagree (1). The scores of the items were added up, so that the overall rating would vary from 6 to 30. Cronbach’s alpha test was estimated at 0.91 for this section.

#### Perceived barriers

This component was represented by 5 items, such as “The lack of traffic signs in the city may cause traffic violations when driving.” The item was measured on a Likert scale ranging from completely agree (5) to completely disagree (1). The scores of the items were added up, so the overall score would range from 5 and 25. For this section, Cronbach’s alpha test was estimated at 0.84.

#### Self-efficacy

There were 4 items included in this component, such as “Safe driving is difficult, but I have the ability to drive safely”. The items were measured on a Likert scale ranging from completely agree (5) to completely disagree (1). The scores of the items were added up, so its score could vary from 4 to 20. Cronbach’s alpha test was estimated at 0.63.

#### Cues to action

This component consisted of 5 items, including “How much do police remind you to drive safely?”. The items were measured on a Likert scale ranging from very much (5) to very little (1). The score of the items were added up, and the overall rating ranged from 5 to 25. Cronbach’s alpha test for this section was estimated at 0.87.

#### Safe driving behaviours

Safe driving behaviours were measured by a questionnaire that was developed by the researchers. This scale included 32 items, for example: “How often do you wear a seatbelt?”, “How often do you drive within the speed limit?” and “How often do you drive between the lines?”. The items were measured on a Likert scale ranging from always (3) to never (0), so the overall score would vary from 0 to 96 and a higher score would represent safer driving behaviours. A pilot study was performed on six taxi drivers who met the inclusion criteria to determine the appropriateness of the questionnaire for the target population. The subjects who participated in the pilot study were asked to respond to questions about the relevance, simplicity and clarity of each item. In this pilot study, no item was omitted, but a few items were rephrased to enhance the clarity and understanding. In addition, the questionnaire was reviewed by four specialists who were experienced in research regarding driving behaviour. Their considerations were then applied to the questionnaire. To test the reliability, the internal consistency was assessed using Cronbach’s alpha coefficient and it was estimated at 0.79.

### Ethics approval

This study was approved by the ethic committee of Hormozgan University of Medical Sciences (Code: HUMS.REC.1396.38). Before data collection the purpose of the study was explained to the participants and informed consent was obtained verbally. Participants at any stage of the research were entitled to withdraw from the study and the researchers did not persuade the participants to stay in the study. Participation in the research did not have any financial burden for the participants. The data collected was anonymous.

### Data analysis

Data were analysed using SPSS ver19. Initially, the score of each HBM construct was calculated independently. A higher score would imply higher susceptibility, severity, benefits and barriers perceived as well as higher self-efficacy and cues to action, all concerning safe driving behaviours. To explore the correlations between the HBM constructs, a Pearson’s correlation coefficient was calculated. To predict the safe taxi driver behaviour using the HBM, multiple regression was conducted.

## Results

The subjects’ demographic profile is presented in Table 1. According to this table, the entire study population was educated, and most of them had a diploma or degree. The majority of the subjects had not received fines for traffic violations while transporting passengers, and a notable number of them had experienced a car accident during their time working as a taxi driver.

**Table 1:** Subjects’ demographic information

Variable	Mean(SD)	Number	Percent
Age (yrs.)	45.1 (11.1)		
Education			
Elementary school		41	22.7
Junior school		47	26.1
High school		23	12.8
Diploma		55	30.6
University degree		14	7.8
Driving experience (yrs.)	18.7 (10.8)		
Riding passengers experience (yrs.)	10.3 (7.5)		
Fines for violation			
Yes		30.6	55
No		69.4	125
Experience of accident (at work)			
Yes		22.2	40
No		77.8	140

The mean and standard deviation of the HBM constructs and safe driving behaviours are presented in Table 2.

**Table 2:** Mean and standard deviation of HBM constructs and safe driving behaviours

Variable	Mean	SD	Range
Perceived susceptibility	23.7	2.5	5–25
Perceived severity	21.5	2.7	5–25
Perceived benefits	28.3	2.9	6–30
Perceived barriers	17.1	5.6	5–25
Self-efficacy	17.3	2.4	4–20
Cues to action	18.9	5.1	5–25
Safe driving behaviours	85.1	6.2	0–96

Among the HBM constructs, cues to action and perceived benefits were shown to be positively correlated with safe driving behaviours, while the perceived barriers were negatively correlated (Table 3).

**Table 3:** Correlation of the HBM constructs

HBM constructs	Perceived susceptibility	Perceived severity	Perceived benefits	Perceived barriers	Self-efficacy	Cues to action
Perceived susceptibility						
Perceived severity	0.12					
Perceived benefits	0.21^*^	0.44^*^				
Perceived barriers	−0.13	0.25^*^	−0.16^*^			
Self-efficacy	0.19^*^	0.31^*^	0.35^*^	0.06		
Cues to action	−0.03	−0.11	0.18	−0.16^*^	0.28^*^	
Safe driving behaviour	0.13	0.11	0.37^*^	−0.31^*^	0.12	0.38^*^

^*^Correlation is significant at *p* < 0.5

Multiple regression analysis revealed that the perceived benefits, perceived barriers and cues to action were predictors of safe driving behaviours (R^2^ = 0.31, F = 12.6, *p*<0.001) (Table 4).

**Table 4:** Linear regression of safe driving behaviours on HBM constructs

Variables	R^2^	B	SE	Beta	*P*
Safe driving behaviours	0.31				
Constant		61.5	5.63		< 0.001*
Perceived susceptibility		0.17	0.16	0.07	0.30
Perceived severity		0.28	0.18	0.12	0.11
Perceived benefits		0.49	0.16	0.23	< 0.004*
Perceived barriers		−0.26	0.08	−0.23	< 0.001*
Self-efficacy		−0.25	0.18	−0.09	0.18
Cues to action		0.43	0.08	0.34	< 0.001*

## Discussion

The present study aimed to predict the taxi drivers’ safe driving behaviours based on the constructs of the HBM in Bandar Abbas. The findings confirmed that cues to action were the strongest predictor of safe driving among the taxi drivers. In their research, Gras et al. observed the significant role of sticker reminders, reminder messages in parking lots, at work and in coffee shops in promoting habitual seatbelt wearing among drivers [38]. Mehri et al. found that the cues to action were the key predictor of safe driver behaviours [39]. Quine et al. reported that cues to action were the primary predictor of adolescent cyclists’ wearing helmets. These researchers emphasized the importance of constantly reminding adolescents to wear helmets in promoting this healthy behaviour [40]. Some other investigations also confirmed the present findings [39, 41–44]. In contrast, Tavafian et al. reported a significant correlation between cues to action and safe behaviour. However, the former was not a strong predictor of the latter [28]. These divergences can be partly explained by different features of the target group, the purpose and the design of the research. On the other hand, in the present study, the target population was taxi drivers, and the different findings may be partly due to warnings from the police, managers, authorities and heads of the taxi driving lines. Similarly, Orouji et al. observed that 61.4% of their research sample perceived awareness-raising as effective in wearing helmets [45]. Another investigation in the U.S. revealed that the majority of drivers did heed prompts in terms of reducing the rate of accidents [46].

The abovementioned literature leads us to conclude that cues to action (external reminders, police constant monitoring) probably plays a key role in road accident-related safe behaviours. Therefore, a number of suggestions have been made to further reduce violations and promote safe behaviours: use of the radio, television, the press, influential people (experienced drivers in each line), motivating messages on bulletin boards, highway billboards, the body of taxis, reminders inside the car especially on the front window, instructive CDs or cassettes (usable while driving) for both the driver and passengers.

Perceived barriers followed cues to action in terms of predicting taxi drivers’ safe behaviours. In the investigations carried out by Orouji et al., the perceived barriers were shown to be the foremost factor in wearing a helmet [45]. Simsekoglu found that perceived barriers were the strongest predictor of wearing seatbelts on city roads [46]. Among the barriers to wearing seatbelts were limits on motion, breathlessness, haste, lethargy and discomfort as reported by Gras et al and Chliaoutakis et al. [38, 47]. Perceived barriers were maintained as being more influential than the perceived benefits and a strong predictor of cyclists’ wearing helmets by Lajunen. They certainly perceived the benefits of wearing helmets but encountered such barriers as the difficulty of carrying one, or simply not being able to afford one. Providing free helmets would to be the most effective strategy in increasing the implementation of this safe riding behaviour [11]. A body of related research also reported similar results [26, 28, 42, 45, 48, 49]. To further increase the rate of safe driving behaviours, these barriers need to be reduced or removed. For example, the terrible physical condition of the roads is a key barrier to safe driving behaviours. It’s likely that the streets are not in good condition. As an example, the street level may not be smooth or the asphalt may not suitable, and has many posts and hills. In some research, taxi drivers were observed to suddenly brake or change directions suddenly to avoid pitfalls, which could threaten the reactions of the cars behind [50]. Among the other barriers to safe driving were limited facilities and existing outdated cars, which significantly lowered the safety level. It, therefore, appears that the existing barriers need to be removed so that safe driving can be further strengthened. Further research and in-depth qualitative interviews are recommended to recognize the other barriers to safe driving and establish safe driving habits.

According to the present findings, the perceived benefits had a significant positive correlation with safe driving habits. Similarly, Quine et al. found that the perceived benefits are the strongest predictor of cyclists’ wearing helmets [40]. However, this was not a good predictor of wearing seatbelts in an investigation by Gras et al [38]. The differing findings may be partly due to differences in study design and purpose, as well as the subjects’ personal and cultural idiosyncrasies. It is also argued that it takes time for some drivers to come to know the benefits of a behaviour and they, therefore, pay less attention. Overall, emphasizing the early perceived benefits rather than the delayed is suggested. The mean score of the perceived benefits in the present study show that the drivers’ perception of the benefits is high in performing safe behaviours. One gradually gets to know that wearing a seatbelt can seriously cut down on probable injuries, which is to one’s own benefit. The perceived benefits were shown to be significantly correlated with safe behaviours in some other investigations [26, 28, 42, 49] . Therefore, it appears that highlighting the benefits through education and raising awareness can dramatically promote safe driving behaviours. To this aim, the Atout-Rout programme was initiated in France to cut down on the rate of accidents among youths. An awareness-raising campaign was held, and the results indicated the effectiveness of this educational intervention on reducing accidents, accident-related death and the mortality and injury rates [51].

The perceived susceptibility was not shown to be a predictor of safe driving behaviours in taxis. However, some other related literature proved that this component was effective in improving safe behaviours [24, 26, 49]. In the present research, the perceived susceptibility was not a predictor of safe behaviours, which is probably due to the target population, i.e., taxi drivers. As taxi drivers often see themselves as highly professional, they do not feel any threat to their life. A study that explored the attitudes of American drivers towards their own driving and that of their peers revealed that the majority of drivers perceived themselves as careful and experienced and were exempt from any risk or threat [50]. However, investigations of other target populations show their susceptibility to accidents. According to the primary version of this theory, some people are inherently more susceptible to unsafe behaviours and, therefore, accidents. A set of features that make one more susceptible to unsafe behaviours compared to others is not stable over time. Once these features change, their susceptibility changes accordingly. This can be explained by their gaining more experience and skills, as well as the debilitation of sensory-motor skills with ageing [52]. Therefore, early prediction and prevention of injuries depends on a true knowledge of the potential and probability of risk. Through raising drivers’ awareness and experience, their perceived susceptibility is also raised, and a set of preventive behaviours emerges as a result.

Perceived severity was not shown to be a predictor of safe driving behaviours. In Lajunenn et al.’s investigation, the perceived severity had minor effects on wearing helmets [11]. Some other research yielded similar results [39]. To support this finding, it can be surmised that the benefits of safe behaviours should be emphasized more than the negative consequences, such as physical injuries, disability and death, to encourage drivers to promote safe behaviours. Being susceptible to risks does not necessarily lead to safe driving behaviours because one does not feel obliged to show a certain behaviour unless s/he feels in danger. Some other investigations disagreed [26, 49]. Perhaps another possibility could be raised. Taxi drivers consider themselves experienced and professional drivers, and since experienced drivers find themselves less at risk, their perceived severity is low.

This disagreement might be due to the different individual and cultural characteristics of target populations, different goals, instruments and the participating subjects. For the individual factors, Strahan determined that violations could be reduced through improving physical health and removing mental pressures. Personality factors, such as extroversion, sensation seeking and cognitive psychological problems, such as depression and anxiety are correlated with high levels of driving violations [53].

The present research showed no significant correlation between self-efficacy and safe driving behaviours. In contrast, other investigations attested to the effectiveness of this construct on safe driving habits [26, 54]. This divergence can be partly explained by the study population in the present research (taxi drivers). Taxi drivers probably perceive themselves capable of pursuing the correct behaviour and quitting when hazardous. They perceive themselves as professionals that are ready to take the right action when needed. It seems logical to search for the roots of any hazardous behaviour from the very outset. Why the target population feels demotivated to follow the right behaviours needs to be explored to promote safe and healthy behaviours.

The present research is limited by the data collection method, i.e., the self-rating design, which might not be adequate. Another limitation is the lack of generalizability of the results. Findings relevant to taxi drivers cannot be generalized to other drivers.

## Conclusions

According to the results of this study, cues to action, perceived benefits and perceived barriers are predictors of safe driving behaviours in taxi drivers**.** In designing health promotion programmes to improve safe driving behaviours among taxi drivers, these constructs should be considered. Therefore, advertising, design of information campaigns, emphasis on the benefits of safe driving behaviours and modification of barriers are recommended. Additionally, it is recommended that police and taxi authorities monitor the driving behaviours of taxi drivers more.

## Abbreviations

HBM: Health Belief Model
TPB: Theory of Planned Behaviour
